# Activated Platelets Autocrine 5-Hydroxytryptophan Aggravates Sepsis-Induced Acute Lung Injury by Promoting Neutrophils Extracellular Traps Formation

**DOI:** 10.3389/fcell.2021.777989

**Published:** 2022-01-17

**Authors:** Yumeng Huang, Qian Ji, Yanyan Zhu, Shengqiao Fu, Shuangwei Chen, Liangmei Chu, Yongfei Ren, Yue Wang, Xuan Lei, Jia Gu, Ningzheng Tai, Dadong Liu

**Affiliations:** ^1^ Department of Burn and Plastic Surgery, Affiliated Hospital, Jiangsu University, Zhenjiang, China; ^2^ Department of Radiation Oncology, Institute of Oncology, Affiliated Hospital, Jiangsu University, Zhenjiang, China; ^3^ Department of General Surgery, Affiliated Hospital, Jiangsu University, Zhenjiang, China; ^4^ Department of Intensive Care Unit, Affiliated Hospital, Jiangsu University, Zhenjiang, China

**Keywords:** sepsis, acute lung injury (ALI), 5-hydroxytryptophan (5-HT), neutrophil extracellular traps (NETs), neutrophil, platelet

## Abstract

Excessive neutrophil extracellular trap (NET) formation is an important contributor to sepsis-induced acute lung injury (ALI). Recent reports indicate that platelets can induce neutrophil extracellular trap formation. However, the specific mechanism remains unclear. *Tph1* gene, which encodes the rate-limiting enzyme for peripheral 5-hydroxytryptophan (5-HT) synthesis, was knocked out in mice to simulate peripheral 5-HT deficiency. Cecal ligation and puncture (CLP) surgery was performed to induce sepsis. We found that peripheral 5-HT deficiency reduced NET formation in lung tissues, alleviated sepsis-induced lung inflammatory injury, and reduced the mortality rate of CLP mice. In addition, peripheral 5-HT deficiency was shown to reduce the accumulation of platelets and NETs in the lung of septic mice. We found that platelets from wild-type (WT), but not *Tph1* knockout (*Tph1* ^*−/−*^), mice promote lipopolysaccharide (LPS)-induced NET formation. Exogenous 5-HT intervention increased LPS-induced NET formation when *Tph1* ^*−/−*^ platelets were co-cultured with WT neutrophils. Therefore, our study uncovers a mechanism by which peripheral 5-HT aggravated sepsis-induced ALI by promoting NET formation in the lung of septic mice.

## Introduction

Sepsis is a life-threatening organ dysfunction caused by the host’s unbalanced response to infection and continues to be a major cause of death resulting from infection (Fleischmann et al., 2016; Rhodes et al., 2017; Napolitano 2018; Xie et al., 2020). The lungs are usually the earliest organ suffering in sepsis (Costa et al., 2006; Wang et al., 2019). Sepsis-induced lung injury is one of the key factors that affect the prognosis of patients with sepsis (Park et al., 2019).

Neutrophils are the most abundant innate immune cells in human blood and constitute the first line of human immunity (Mantovani et al., 2011; Wang and Chen, 2018; Abrams et al., 2019). In the past years, it was believed that neutrophils kill bacteria through phagocytosis and degranulation (Kolaczkowska and Kubes, 2013). In 2004, a new mechanism of neutrophil bactericidal function was discovered by Brinkmann et al. (2004), namely, neutrophil extracellular traps (NETs). Subsequently, NETs were considered to be a protective mechanism through capturing and eradicating pathogens (Jenne et al., 2013a; Amulic et al., 2017; Knackstedt et al., 2019; Drury et al., 2021). With the continuous research on NETs, some investigators indicated that excessive NET formation was an important cause of sepsis-induced organ dysfunction and death (Sonego et al., 2016; Ravindran et al., 2019; Tan et al., 2020). Sepsis-induced acute lung injury (ALI) can also be accentuated by NETs (Jhelum et al., 2018; Wang et al., 2020; Yaqinuddin and Kashir, 2020). However, the mechanism still remains unclear.

Serotonin, also named 5-hydroxytryptophan (5-HT), acts as a neurotransmitter in the central nervous system and enteric nervous system (McLean et al., 2007; Brommage et al., 2015). In peripheral blood, 5-HT is synthesized by tryptophan hydroxylase 1 (TPH1) in enterochromaffin cells and mainly stored in dense granules of platelets (Mohammad-Zadeh et al., 2008). Accumulating studies have depicted the critical role of peripheral 5-HT in inflammatory response (Duerschmied et al., 2013; Li et al., 2016; Zhang et al., 2017). However, whether peripheral 5-HT involves in lung NET formation in sepsis remains unclear.

Our data reveal that peripheral 5-HT deficiency can protect against sepsis-induced lung injury and improve the survival rate of septic mice by inhibiting the formation of NETs in lung tissues. This is fundamental to the development of measures to protect organs from injury and dysfunction in sepsis.

## Materials and Methods

### Materials

Lipopolysaccharide (LPS), 5-HT, fetal bovine serum (FBS), bovine serum albumin (BSA), and Tyrode’s solution were obtained from Sigma-Aldrich (MO, United States). ELISA kits for tumor necrosis factor alpha (TNF-α) and interleukin 6 (IL-6) were obtained from Qiaoyi (Shanghai, China). HBSS and RPMI 1640 medium were obtained from Life Technologies (CA, United States). APC-Cy7-labeled anti-LY6G antibody and FITC-labeled anti-CD41 antibody were obtained from BD (NJ, United States). APC-labeled anti-CD62p antibody (p-selectin) was obtained from BioLegend (San Diego, United States). Rabbit anti-mouse citrullinated histone H3 (CitH3) was obtained from Abcam (Cambridge, United Kingdom). Rabbit anti-mouse Ly6G, rabbit anti-mouse CD41, and related fluorescent secondary antibodies were obtained from Cell Signaling Technology (MA, United States).

### Animal Model of Cecal Ligation and Puncture

Wild-type (WT) and *Tph1* knockout (*Tph1* ^*−/−*^) C57BL/6 male mice (6–8 weeks, body weight 20 ± 2 g) were purchased from the Model Animal Research Center of Nanjing University (Nanjing, Jiangsu, China) and raised in the Experimental Animal Center of Jiangsu University (Zhenjiang, Jiangsu, China). All mice were randomly divided into four groups, including the WT and *Tph1* ^*−/−*^ sham groups that received sham surgery as well as the WT and *Tph1* ^*−/−*^ cecal ligation and puncture (CLP) groups that underwent CLP surgery. During CLP surgery, the mice were anesthetized with sevoflurane inhalation, the abdomen was disinfected with alcohol, and a 1-cm midline abdominal incision was made to expose the cecum of the mice. The end 1/3 of the cecum was ligated with 3–0 nylon thread, the end cecum was punctured with a 21-gauge needle, and an appropriate amount of intestinal content spilled out from the perforation. After the treatment, the cecum was reset, and the abdominal incision of the mice was sutured. Sham surgery requires only the cecum to be properly turned over and exposed, and then, the cecum is reset and the abdominal incision is sutured layer by layer. For survival analysis, in the sham groups (WT and *Tph1* ^*−/−*^), each had six mice, while in CLP groups (WT and *Tph1* ^*−/−*^), each had 25 mice. For other experiments, there were four mice in each group. The experimental protocol on animal protection and welfare was approved by the Council on Animal Care and Use at Jiangsu University.

### Neutrophil and Platelet Isolation

Neutrophils were isolated from WT mice bone marrow by using density gradient centrifugation as previously described (Boxio et al., 2004; Szatmary et al., 2017). WT mice were sacrificed. Bone marrow was extracted from the femurs and tibias of the mice. Marrow cells were harvested from the bone marrow with a 70-µm cell strainer. Then, the cells were pelleted in a centrifuge and resuspended in HBSS. Following erythrocyte lysis, the neutrophil-containing solution was placed onto a discontinuous Percoll gradient solution (Percoll solution diluted to 78%, 69%, and 52% in HBSS), and the gradient was centrifuged at 1,500*g* at room temperature for 30 min. Neutrophils were collected from a band between the 78% and 69% layers. Finally, the neutrophils were resuspended in RPMI 1640 with 1% heat-inactivated FBS.

Platelets were isolated from WT and *Tph1* ^*−/−*^ mice peripheral blood as previously described (Liu et al., 2013). Mice peripheral blood was collected into a vacuum-anticoagulated tube containing trisodium citrate, and Tyrode’s solution was added. Platelet-rich plasma were isolated by centrifuging at 180×*g* at room temperature for 10 min, and platelets were isolated by centrifuging at 1,250×*g* at room temperature for 10 min. Then, platelets were washed and resuspended in Tyrode’s solution for at least 1 h at 37°C before use.

The purity (>97%) of cells was detected and adjusted by flow cytometry and an APC-Cy7-labeled anti-LY6G antibody (neutrophil) and FITC-labeled anti-CD41 antibody (platelet). The cell concentration was maintained at 1 × 10^6^/ ml at every experiment. The activity of platelets was detected by flow cytometry and an APC-labeled anti-CD62p antibody.

### Cell Culture and Stimulation

WT neutrophils were randomly divided into three groups, including the WT neutrophil group, WT platelet and WT neutrophil co-culture group, and *Tph1* ^*−/−*^ platelet and WT neutrophil co-culture group. In each group, cells were randomly divided into four groups, with each containing 4 × 10^5^ cells, including a control group, which received no intervention; an LPS group, which received LPS (1 μg/ ml) stimulation; a 5-HT group, which received 5-HT (100 μM) intervention; and an LPS + 5-HT group, which received 5-HT (100 μM) intervention and LPS (1 μg/ ml) stimulation. Cells were incubated in a 5% CO_2_ incubator at 37°C and 95% humidity. Twelve hours later, cells were harvested for subsequent experiments.

### Immunofluorescence of NET Formation and Quantification of NET Formation

Immunofluorescence was used to detect the NET formation in the lung of mice and *in vitro* cultured cells. Lung tissues were sequentially fixed by 4% paraformaldehyde, embedded in paraffin, sectioned, and permeabilized with 0.05% Triton X-100. Cells were harvested, fixed, and permeabilized. Then, both lung and cell specimens were stained with primary antibodies (1:200), including citH3, Ly6G, and/or CD41, overnight at 4°C. Fluorescent secondary antibodies (1:200) were added and incubated at room temperature for 1 h in the dark. DAPI (1 μm/ ml) was incubated in the dark for 15 min. Finally, NET formation was detected by using an inverted phase-contrast microscope (×400 magnification). NETs expression was calculated based on CitH3 staining.

### Histopathological Examination of Mouse Lungs

Lung tissue specimens (approximately 0.4 g) were harvested 12 h after surgery and fixed in 10% formalin. Then, the fixed specimens were sequentially embedded in paraffin, sectioned, and stained with hematoxylin/eosin (HE). A light microscope (×400 magnification) was applied to examine alveolar structure, cellular edema, and granulocyte infiltration in lung specimens.

### TNF-α and IL-6 in Mice Bronchoalveolar Lavage Fluid

Mice were sacrificed, and endotracheal intubation was performed 12 h after surgery. Bronchoalveolar lavage fluid (BALF) was collected from the endotracheal tube by lavage with PBS without Ca^2+^ and Mg^2+^. ELISA kits were used to detect the level of TNF-α and IL-6.

### Survival Rate of Mice

A total of 62 mice were randomly divided into four groups: WT sham group (*n* = 6), WT CLP group (*n* = 25), *Tph1* ^*−/−*^ sham group (*n* = 6), and *Tph1* ^*−/−*^ CLP group (*n* = 25). All mice were raised in the same environment, with no restrictions on their food or water intake. The mice were monitored every 6 h for 72 h.

### Statistics

Statistical analyses were performed with GraphPad Prism version 9.0 (United States). One-way analysis of variance was used for comparison between multiple groups, *t*-test was used for comparison between two groups, and Dunnett’s test was used for *post-hoc* analysis comparison. All data are presented as mean ± SD. Survival rate analysis was performed using the Kaplan–Meier method. A *p* value <0.05 was considered to be statistically significant.

## Results

### Peripheral 5-HT Deficiency Improves Survival Rate of Septic Mice and Alleviates Sepsis-Induced Acute Lung Injury

Peripheral 5-HT, a monoamine neurotransmitter that is mainly synthesized by TPH1, can promote the inflammatory response by activating immune cells and increasing inflammatory cytokine release (Duerschmied et al., 2013; Li et al., 2016; Zhang et al., 2017). TPH1 is a rate-limiting enzyme for the synthesis of peripheral 5-HT (Zhang et al., 2020). In order to explore the role of peripheral 5-HT in sepsis, *Tph1* ^*−/−*^ mice were constructed and CLP was performed to induce sepsis. As shown in Supplementary Figure S1, the 5-HT concentration was markedly reduced in platelets of *Tph1* ^*−/−*^ mice, which confirmed the functional deficiency of TPH1.

**FIGURE 1:**
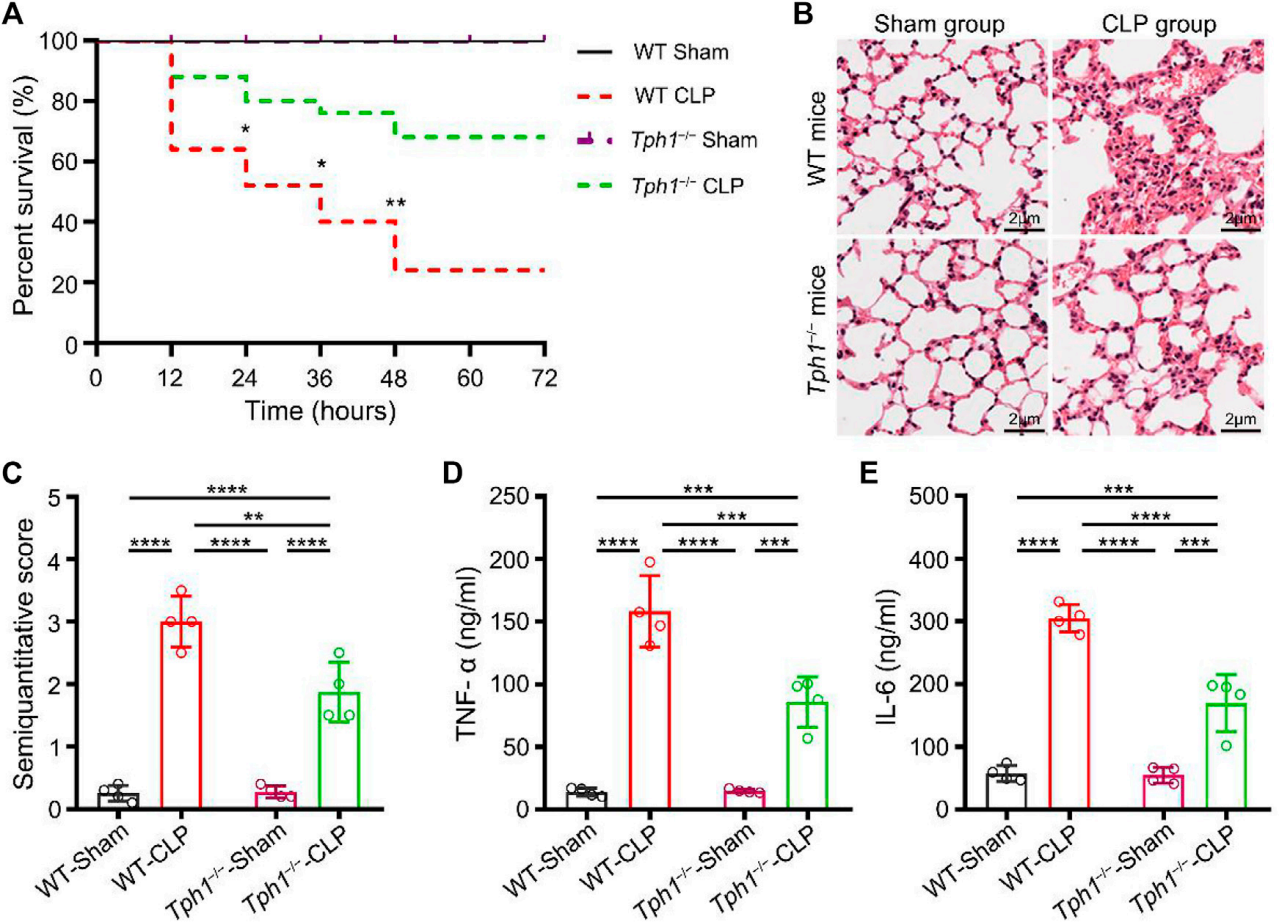
Peripheral 5-hydroxytryptophan (5-HT) deficiency improves the survival rate of septic mice and alleviates sepsis-induced acute lung injury. **(A)** The survival rate of mice. No deaths occurred in the wild-type (WT) and *Tph1* knockout (*Tph1* ^*−/−*^) sham groups. Most of the mice challenged with CLP died between 12 and 24 h postoperatively. The survival rate of WT mice was substantially low (only 52.00% at 24 h). In contrast, the survival rate of *Tph1* ^*−/−*^ mice was significantly increased (80.00% at 24 h). Moreover, at 72 h postoperatively, peripheral 5-HT deficiency dramatically increased the survival of septic mice from 24.00% to 68.00% (*p *< 0.01). For WT and *Tph1* ^*−/−*^ sham groups: *n* = 6; for WT and *Tph1* ^*−/−*^ cecal ligation and puncture (CLP) groups: *n* = 25. **(B)** Histopathological changes. Lung specimens from the sham mice showed normal architectures and lesser granulocyte infiltration, while lung specimens from WT CLP mice showed severe alveolar structure destruction, hyperemia, thickening of alveolar walls, and extensive granulocyte infiltration. On the contrary, lung specimens from the *Tph1* ^*−/−*^ CLP mice group showed less severe alveolar structure destruction and mild alveolar wall granulocyte infiltration. **(C)** Semiquantitative score of lung histological injury. Semiquantitative scores in WT CLP mice were significantly increased compared to sham groups. In contrast, *Tph1* ^*−/−*^ CLP mice showed lower semiquantitative scores. **(D)** Tumor necrosis factor alpha (TNF-α) concentrations. TNF-α concentrations in bronchoalveolar lavage fluid (BALF) of WT CLP mice were significantly increased compared to sham groups. In contrast, *Tph1* ^*−/−*^ CLP mice showed lower TNF-α concentrations. **(E)** Interleukin 6 (IL-6) concentrations. IL-6 concentrations in BALF of WT CLP mice were significantly increased compared to sham groups. In contrast, *Tph1* ^*−/−*^ CLP mice showed lower IL-6 concentrations. For each group: *n* = 4 **(B–E)**. *p < 0.05, **p < 0.01, ***p < 0.001, and ****p < 0.0001.

The effect of peripheral 5-HT on the survival rate of septic mice was examined postoperatively. Results showed that peripheral 5-HT deficiency significantly increased the survival of septic mice (Figure 1A). Further study showed that in the *Tph1* ^*−/−*^ CLP mice, alveolar structure injury was less severe and the extent of alveolar wall granulocyte infiltration was reduced (Figures 1B, C). Meanwhile, we found that *Tph1* ^*−/−*^ CLP mice had a minor increase in BALF TNF-α and IL-6. The differences were statistically significant when compared to WT CLP mice (Figures 1D, E). These results suggested that peripheral 5-HT deficiency improves the survival rate of septic mice and alleviates lung inflammatory injury.

### Peripheral 5-HT Deficiency Inhibits NET Formation in the Lung of Septic Mice

**FIGURE 2:**
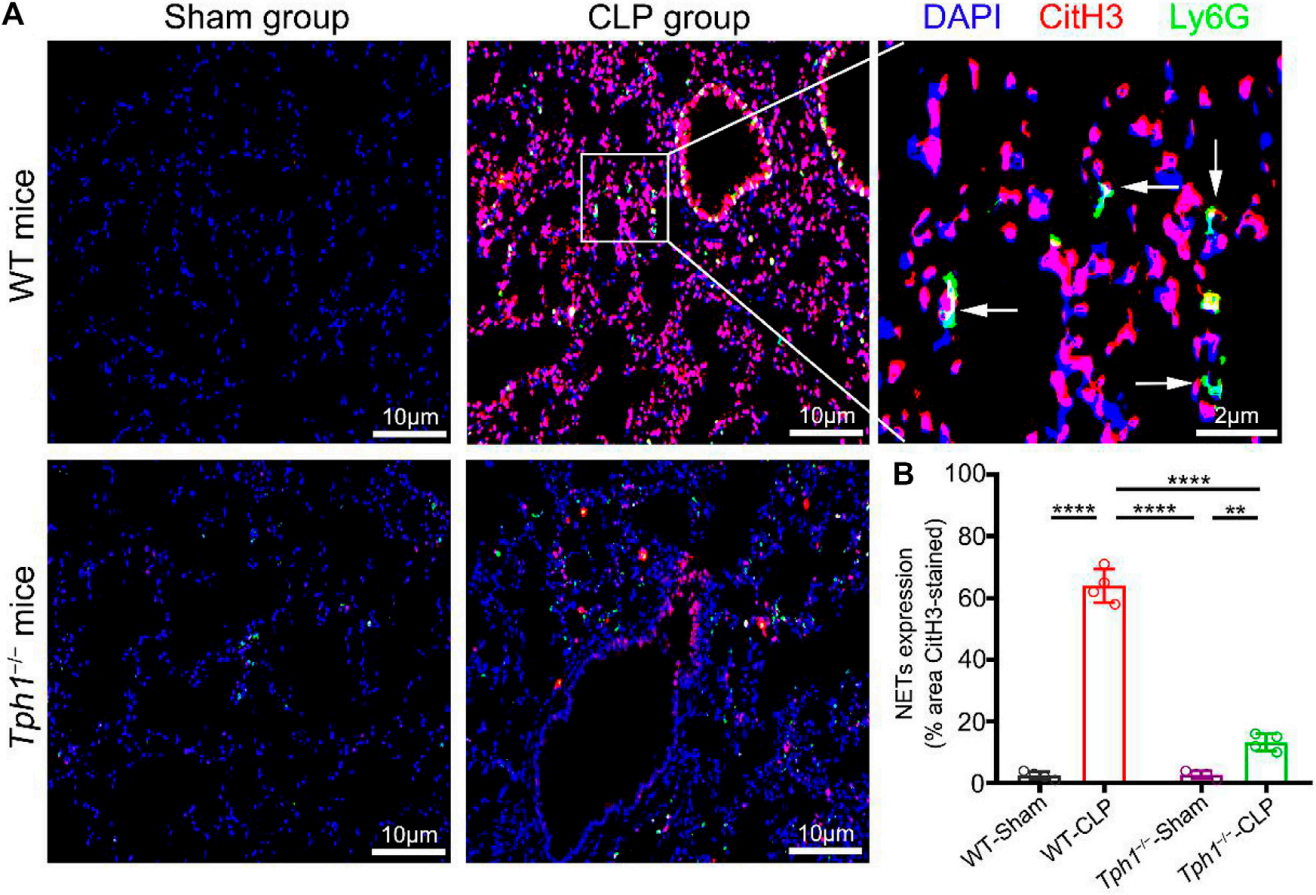
Peripheral 5-HT deficiency inhibits neutrophil extracellular trap (NET) formation in the lung of septic mice. Both WT and *Tph1* ^*−/−*^ mice (male, 6–8 weeks) were selected and randomly divided into sham and CLP groups. The mice were sacrificed 12 h after surgery, and lung specimens were collected and co-stained with Ly6G, CitH3, and DAPI fluorescent antibodies. The accumulation of NETs was detected by immunofluorescence microscopy. **(A)** Representative images of NET formation in the lung of septic mice. **(B)** Analysis of NET expression in mice lung specimens. For each group: *n* = 4. *p < 0.05, **p < 0.01, ***p < 0.001, and ****p < 0.0001.

Uncontrolled NET formation is reported to be a major contributor to sepsis-induced ALI and acute respiratory distress syndrome (ARDS) (Jhelum et al., 2018; Wang et al., 2020; Yaqinuddin and Kashir, 2020). Lefrancais et al. (2018) showed that inhibiting NET formation could reduce lung injury and improve mice survival. Lung specimens were collected, and immunofluorescence studies were performed to determine whether peripheral 5-HT deficiency affected NET formation. Results showed that lung tissues from sham mice had no NET formation. However, lung tissues from WT CLP mice had significantly enhanced NET formation, as indicated by the staining of CitH3. Interestingly, we observed that in the lung tissues of *Tph1* ^*−/−*^ CLP mice, NET formation was significantly reduced (Figure 2). This result indicates that peripheral 5-HT deficiency can reduce NET formation in the lung of septic mice.

### Peripheral 5-HT Deficiency Inhibits the Accumulation of Platelets With NETs in the Lung of Septic Mice

**FIGURE 3:**
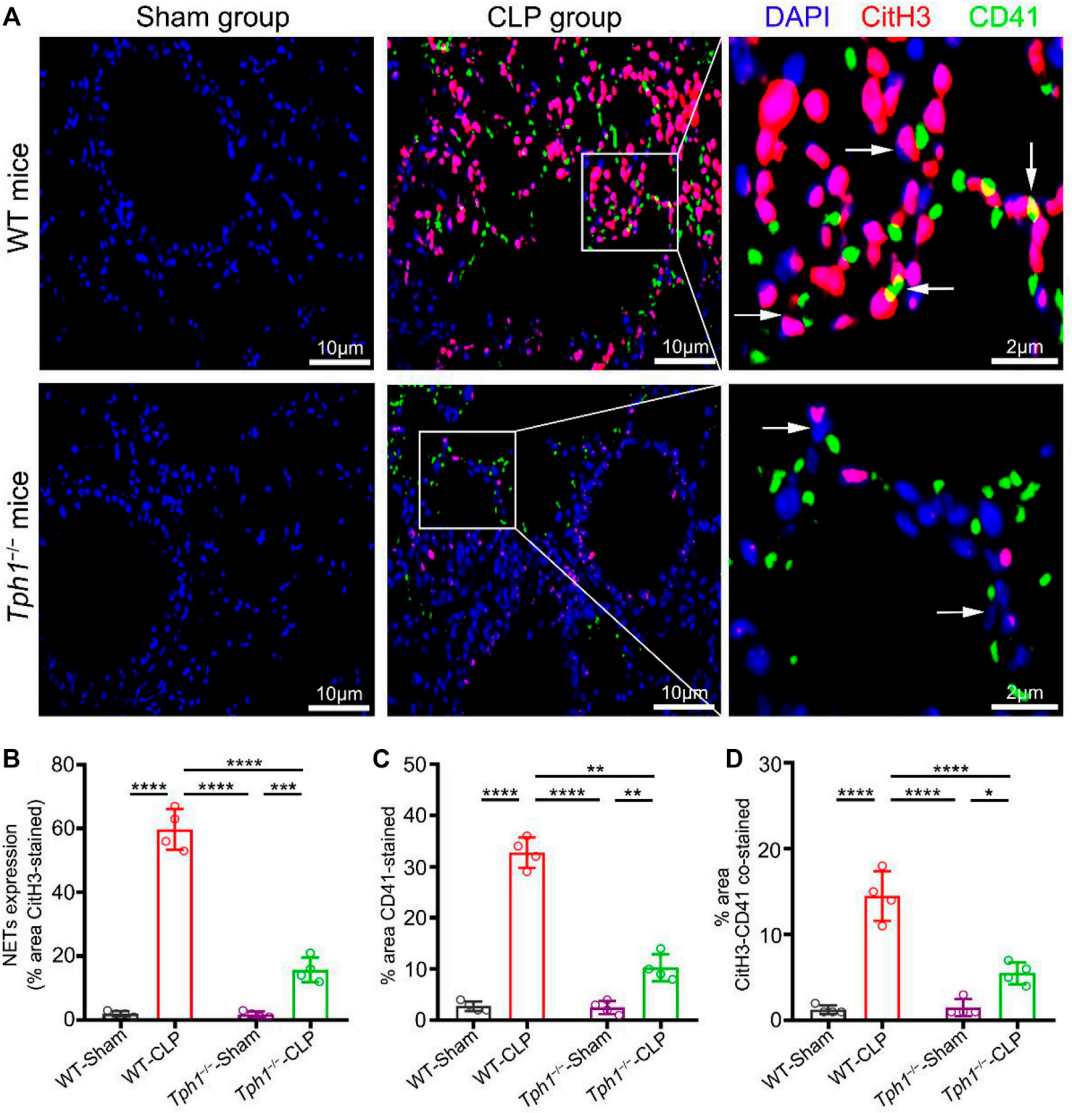
Peripheral 5-HT deficiency inhibits the accumulation of platelets with NETs in the lung of septic mice. Both WT and *Tph1* ^*−/−*^ mice (male, 6–8 weeks) were selected and randomly divided into sham and CLP groups. The mice were sacrificed 12 h after surgery, and lung specimens were collected and co-stained with CD41, CitH3, and DAPI fluorescent antibodies. The accumulation of NETs was detected by immunofluorescence microscopy. Lung specimens from sham mice showed little accumulation of platelets and NETs. Lung specimens from WT CLP mice group showed a large number of accumulation of platelets and NETs, while lung specimens from *Tph1* ^*−/−*^ CLP mice showed a significant decrease of the accumulation of platelets and NETs. **(A)** Representative images of the accumulation of platelets and NETs in the lung of septic mice. **(B)** Analysis of NET expression in mice lung specimens. **(C)** Analysis of the percentage of CD41-stained lung specimens in mice. **(D)** Analysis of the percentage of CitH3–CD41-stained lung specimens in mice. For each group: *n* = 4. *p < 0.05, **p < 0.01, ***p < 0.001, and ****p < 0.0001.

Peripheral 5-HT is mainly stored in dense granules of platelets and released into the plasma during platelet activation (Mohammad-Zadeh et al., 2008). In order to investigate whether platelet-derived 5-HT is associated with NET formation, lung specimens were collected and co-stained with anti-CitH3 (labeled NETs) and anti-CD41 (labeled platelets). Results showed that the lungs from sham mice did not show NET formation, while the lungs from WT CLP mice showed a mass of the accumulation of platelets and NETs. Interestingly, the lungs from *Tph1* ^*−/−*^ CLP mice showed a significant decrease of the accumulation of platelets and NETs (Figure 3). We conclude that peripheral 5-HT deficiency can reduce the accumulation of platelets and NETs in the lung of septic mice.

### Activated Platelets Promote NET Formation Through Autocrine 5-HT

**FIGURE 4:**
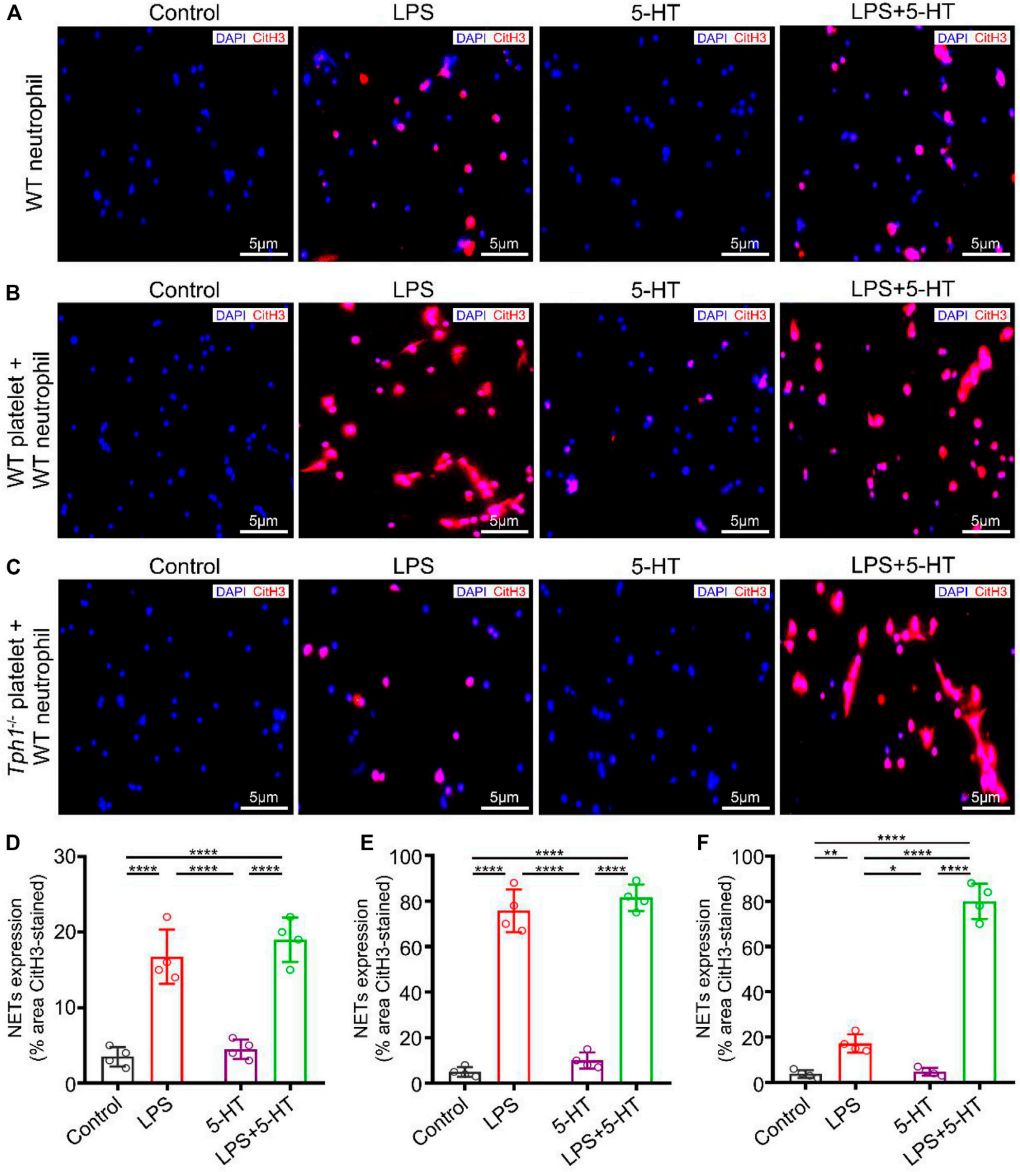
Activated platelets promote NET formation through autocrine 5-HT. Neutrophils were isolated from WT mice. Platelets were isolated from WT and *Tph1* ^*−/−*^ mice. The WT neutrophils were divided into WT neutrophil culture alone, WT neutrophil co-culture with WT platelet, and WT neutrophil co-culture with *Tph1* ^*−/−*^ platelet groups. In each group, cells were randomly divided into control, lipopolysaccharide (LPS), 5-HT, and 5-HT + LPS subgroups. After 12 h of cultivation, cells were fixed and co-stained with CitH3 fluorescent antibodies and DAPI. The accumulation of NETs was detected by immunofluorescence microscopy. Representative images of NET formation are presented in this figure. **(A)** Representative images of NET formation in WT neutrophils cultured alone. **(B)** Representative images of NET formation in WT neutrophils co-cultured with WT platelets. **(C)** Representative images of NET formation in WT neutrophils co-cultured with *Tph1* ^*−/−*^ platelets. **(D)** Analysis of NET expression in WT neutrophils. **(E)** Analysis of NET expression in WT neutrophils co-cultured with WT platelets. **(F)** Analysis of NET expression in WT neutrophils co-cultured with *Tph1* ^*−/−*^ platelets. For each group: *n* = 4. *p < 0.05, **p < 0.01, ***p < 0.001, and ****p < 0.0001.

Although accumulating evidences reveal the role of platelets in NET formation, its mechanisms is unknown. To address this issue, neutrophils were isolated from WT mice and stimulated by LPS with or without exogenous 5-HT *in vitro*. NET generation was detected by staining with anti-CitH3 and DAPI. We noticed that LPS stimulation induced NET formation, while exogenous 5-HT intervention did not increase the LPS-induced NET formation in WT neutrophils cultured alone (Figures 4A, D). Recent studies showed that in bacterial sepsis, LPS induces non-classical activation of platelets (Clark et al., 2007; Carestia et al., 2016; Martinod and Deppermann, 2021). Consistent with the above studies, our mean fluorescence intensity (MFI) result showed that P-selectin (also called CD62p—a platelet activation marker) (van Velzen et al., 2012) was highly expressed in LPS-stimulated platelets. Furthermore, the MFI of P-selectin in WT platelets was higher than that in *Tph1* ^*−/−*^ platelets (Supplementary Figure S2). To further investigate whether platelet autocrine 5-HT accounted for NET formation, LPS was used to activate platelets. Platelets were isolated from WT or *Tph1* ^*−/−*^ mice and co-incubated with the WT neutrophils. Interestingly, results showed that WT platelets promoted LPS-induced NET formation (Figures 4B, E), while *Tph1* ^*−/−*^ platelets did not show a promotive effect (Figures 4C, F). However, exogenous 5-HT intervention remedied the deficiency of *Tph1* ^*−/−*^ platelets and promoted LPS-induced NET formation (Figures 4C, F). In light of the results shown above, we conclude that activated platelets promote NET formation through autocrine 5-HT signaling.

## Discussion

NETs are extracellular strands of decondensed DNA that are decorated with histones and neutrophil granule proteins. Since the discovery of NETs, numerous studies on NETs have been published. Among these findings, one widely accepted fact is that NETs may function as a double-edged sword (Kaplan and Radic, 2012). First of all, NET formation plays a central role in antimicrobial immunity. It constitutes an effective antimicrobial defense by neutralizing and killing pathogens at the infected site (Brinkmann et al., 2004; Papayannopoulos, 2018). On the other hand, NETs and their components may amplify the inflammatory process and promote organ damage, especially in non-infected organs of sepsis (Folco et al., 2018; Denning et al., 2019). In addition, detection of circulating NET components (including myeloperoxidase-DNA and citrullinated histone H3) can be used to assess organ impairment and predict 28-day mortality rate in septic patients (Li et al., 2011; Maruchi et al., 2018; Nomura et al., 2019). Consistent with previous reports, our results also revealed that the NET formation in lung tissue is associated with lung inflammatory injury and high mortality of CLP mice. Therefore, further studies were performed to explore the specific mechanism of sepsis-induced NET formation in the lungs.

The 5-HT receptor is classically recognized as a neurotransmitter (Okaty et al., 2019). Recently, the pro-inflammatory effect of platelet-derived 5-HT has attracted extensive attention (Duerschmied et al., 2013; Wu et al., 2019). Cloutier et al.,(2012) found that inhibiting the uptake of 5-HT in platelets could reduce joint effusion during arthritis. In recent years, studies have shown that the severity of pneumonia is also related to the tryptophan/serotonin pathway (Meier et al., 2017). Also, 5-HT can increase the exudation of neutrophils during ALI, which is believed to be related to the recruitment of neutrophils in innate immunity (Duerschmied et al., 2013). Our *in vivo* studies confirmed that peripheral 5-HT deficiency protects the lungs of mice from sepsis-induced lung injury and at the same time reduces NET formation in the lung tissues. However, in further *in vitro* experiments, we found that exogenous addition of 5-HT could not directly affect the NET formation induced by LPS. Therefore, we further explored its potential mechanisms.

In the lungs of septic mice, we found that platelets were accumulated with NETs, which suggested that platelets might have contributed to the NET formation in septic lungs. Platelets are tiny disk-shaped anucleate cell fragments derived from bone marrow megakaryocytes that play central roles in thrombosis and inflammation (Weiss, 1975; Jenne et al., 2013b; Deppermann and Kubes, 2018). Platelets contain three types of granules: α-granules, dense granules, and lysosomes. When platelets are activated, these granules can release a variety of secretions (Koupenova et al., 2018). Recent studies have shown that platelets are involved in NET formation (Clark et al., 2007). Meanwhile, platelet-promoted NET formation is associated with inflammation and thrombosis (Papayannopoulos, 2018; Mauler et al., 2019; Martinod and Deppermann, 2021). Similar, in our *in vitro* study, we also found that WT mice-derived platelets activated by LPS could significantly promote NET formation. However, the specific mechanism of platelet-promoted NET formation is still controversial.

Peripheral 5-HT is mainly stored in platelets and released after platelets are activated (Walther et al., 2003; Mammadova-Bach et al., 2018). The platelet autocrine 5-HT can further activate platelets (Li et al., 1997). In our study, we found that the activated *Tph1* ^*−/−*^ platelets (lacking 5-HT) did not increase NET formation, while the exogenous addition of 5-HT intervention can effectively reverse this phenomenon. These evidences suggest that platelet autocrine 5-HT plays a promoter role in LPS-induced NET formation. P-selectin can promote NET formation by mediating the binding of platelets to neutrophils (Pircher et al., 2019). However, it is still controversial whether P-selectin participated into the platelet-promoted NET formation (Maugeri et al., 2014; Etulain et al., 2015). In our study, we found that compared with *Tph1* ^−/−^ platelets, LPS could induce P-selectin expression in WT platelets more strongly. It indicates that platelet autocrine 5-HT-induced NET formation may involve P-selectin-mediated platelet and neutrophil interaction. Of course, we cannot completely ignore that other secretions may also have certain effects.

In conclusion, our findings indicate that platelet activation promotes NET formation in the lung tissue of septic mice through autocrine 5-HT signaling. Peripheral 5-HT deficiency protects the lung from sepsis-induced injury and improves the survival rate of septic mice by inhibiting the NET formation in lung tissues. This study is only conducted in animal and cell experiments and did not further explore the pathway through which activated platelet autocrine 5-HT acted on platelets to increase NET formation; further exploration and further clinical studies are warranted.

## Data Availability

The raw data supporting the conclusions of this article will be made available by the authors, without undue reservation.
