# Supplementary material for: Activated Platelets Autocrine 5-Hydroxytryptophan Aggravates Sepsis-Induced Acute Lung Injury by Promoting Neutrophils Extracellular Traps Formation
Source: Front Cell Dev Biol. 2022 Jan 17;9:777989. doi: 10.3389/fcell.2021.777989 (PMC8801939; doi:10.3389/fcell.2021.777989)
Supplement: Supplementary file 1 [file DataSheet1.docx]

Supplementary Material


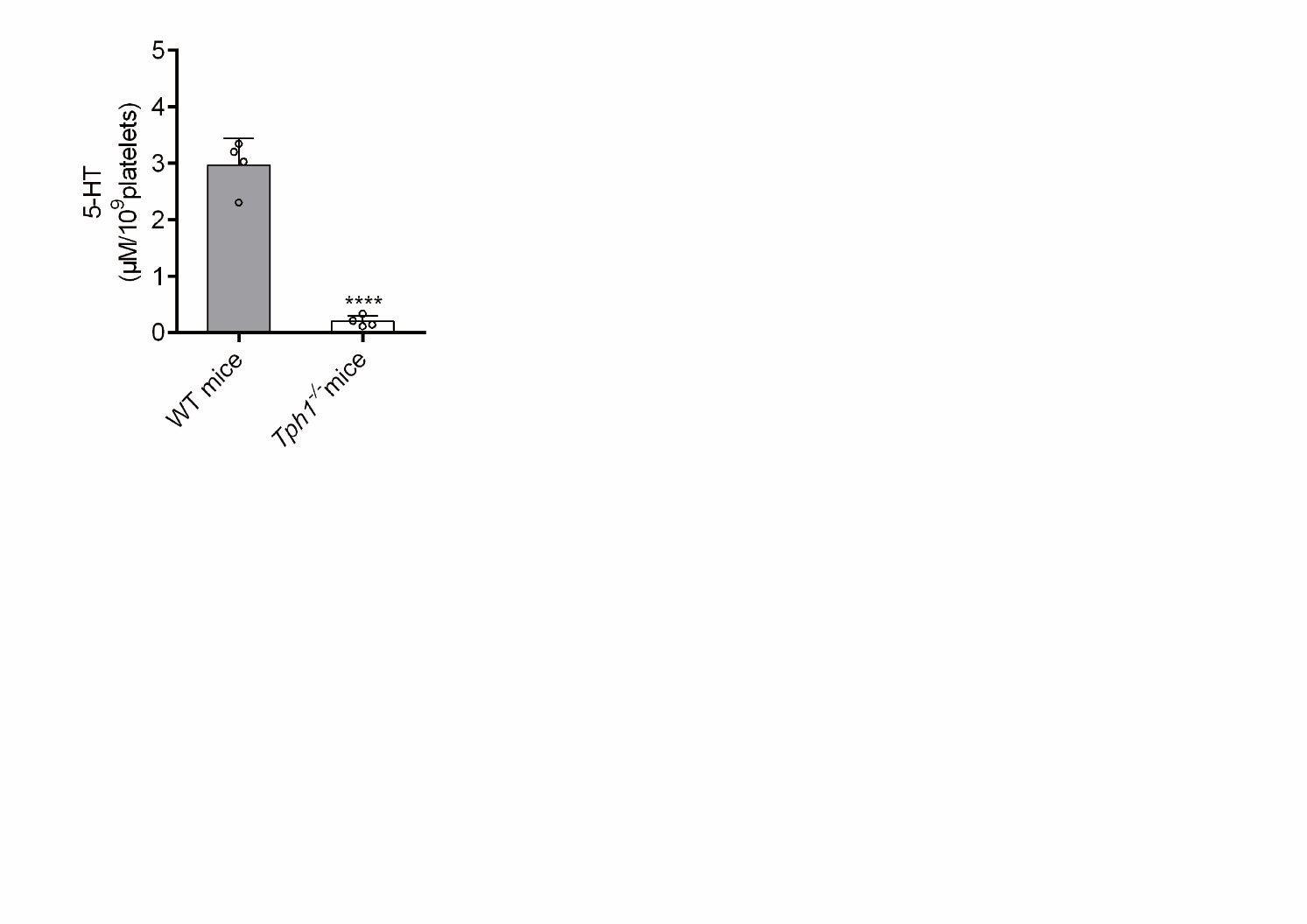


**Supplementary Figure 1.** **5-HT in platelets of *Tph1^-/-^* mice was significantly reduced compared with WT mice.** 5-HT in platelets of WT mice and *Tph1^-/-^* mice. For each group: n=4. **P* < 0.05, ***P* < 0.01, ****P* < 0.001, *****P* < 0.0001.


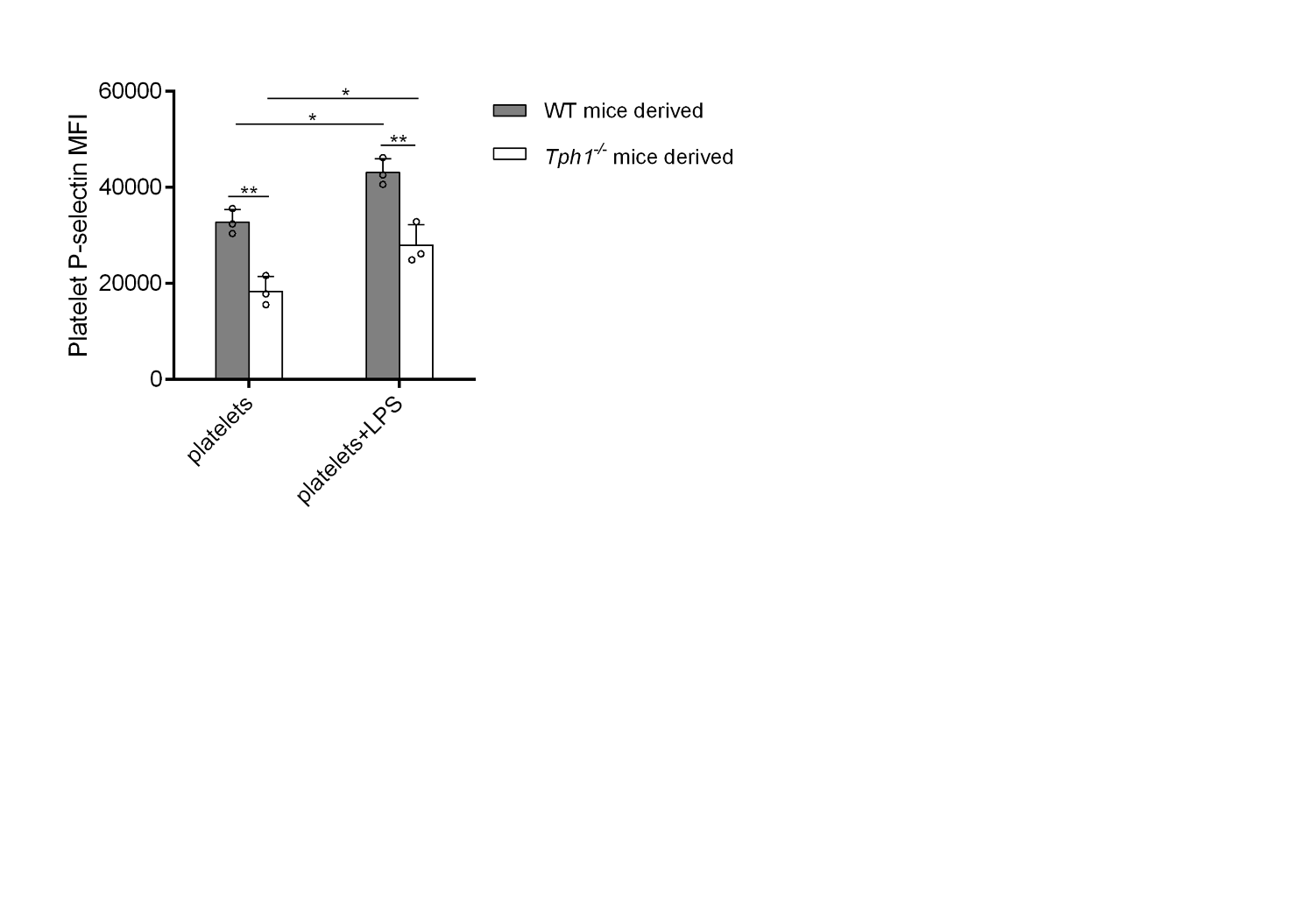


**Supplementary Figure 2. The activity of platelets from WT mice was higher than that from *Tph1^-/-^* mice with LPS-induced or not.** Mean fluorescence intensity (MFI) of WT and *Tph1^-/-^* mice derived platelets P-selectin (CD62p) with LPS-induced or not. For each group: n=3. **P* < 0.05, ***P* < 0.01, ****P* < 0.001, *****P* < 0.0001.
